# Supplementary figures and images for: Comparison and Optimization of Continuous Flow Reactors for Aerobic Granule Sludge Cultivation from the Perspective of Hydrodynamic Behavior
Source: Int J Environ Res Public Health. 2022 Jul 7;19(14):8306. doi: 10.3390/ijerph19148306 (PMC9320295; doi:10.3390/ijerph19148306)

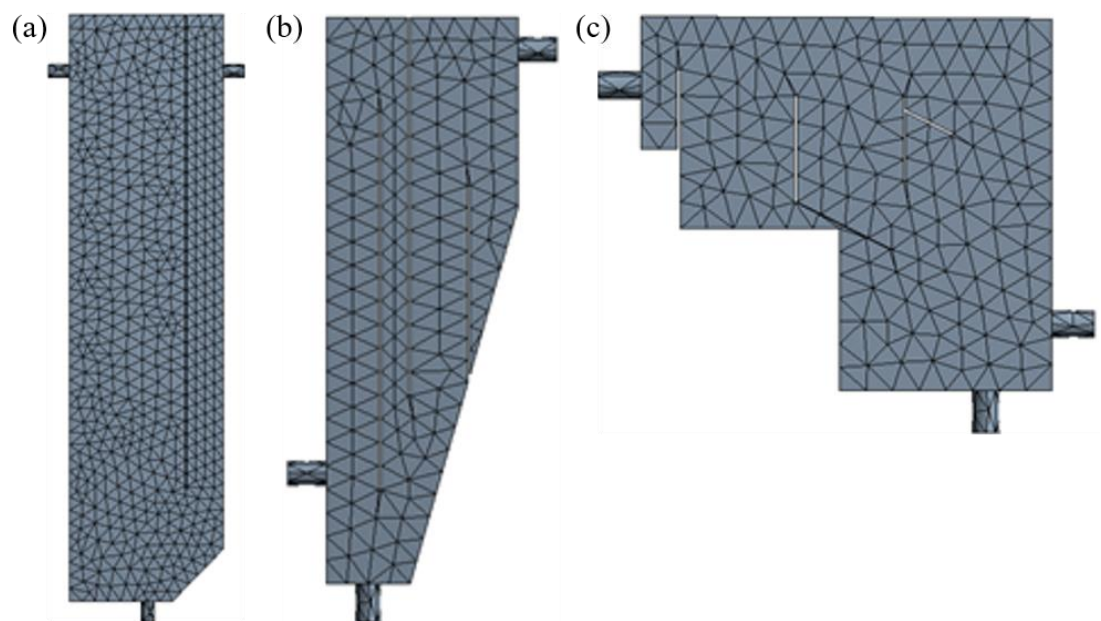

Fig. S1 The mesh of R1(a), R2(b) and R3(c)

Supplement: Supplementary file 1 [file ijerph-19-08306-s001.zip › ijerph-1750556-supplementary.pdf]
